# A Contact Mechanics Model for Surface Wear Prediction of Parallel-Axis Polymer Gears

**DOI:** 10.3390/polym16202858

**Published:** 2024-10-10

**Authors:** Enis Muratović, Nedim Pervan, Adil Muminović, Muamer Delić

**Affiliations:** Department of Mechanical Design, Faculty of Mechanical Engineering, University of Sarajevo, 71000 Sarajevo, Bosnia and Herzegovina; muratovic@mef.unsa.ba (E.M.); muminovic@mef.unsa.ba (A.M.); delic@mef.unsa.ba (M.D.)

**Keywords:** surface wear, contact mechanics model, boundary element method, Archard’s wear equation, wear coefficient, Winkler’s surface formulation, Weber’s model, load-sharing factor, normal load distribution, experimental testing

## Abstract

As surface wear is one of the major failure mechanisms in many applications that include polymer gears, lifetime prediction of polymer gears often requires time-consuming and expensive experimental testing. This study introduces a contact mechanics model for the surface wear prediction of polymer gears. The developed model, which is based on an iterative numerical procedure, employs a boundary element method (BEM) in conjunction with Archard’s wear equation to predict wear depth on contacting tooth surfaces. The wear coefficients, necessary for the model development, have been determined experimentally for Polyoxymethylene (POM) and Polyvinylidene fluoride (PVDF) polymer gear samples by employing an abrasive wear model by the VDI 2736 guidelines for polymer gear design. To fully describe the complex changes in contact topography as the gears wear, the prediction model employs Winkler’s surface formulation used for the computation of the contact pressure distribution and Weber’s model for the computation of wear-induced changes in stiffness components as well as the alterations in the load-sharing factors with corresponding effects on the normal load distribution. The developed contact mechanics model has been validated through experimental testing of steel/polymer engagements after an arbitrary number of load cycles. Based on the comparison of the simulated and experimental results, it can be concluded that the developed model can be used to predict the surface wear of polymer gears, therefore reducing the need to perform experimental testing. One of the major benefits of the developed model is the possibility of assessing and visualizing the numerous contact parameters that simultaneously affect the wear behavior, which can be used to determine the wear patterns of contacting tooth surfaces after a certain number of load cycles, i.e., different lifetime stages of polymer gears.

## 1. Introduction

The rapid growth of polymer materials and continuously increasing demands for lightweight components have led to the increased use of polymer gears, as one of the main components in power transmission, in many engineering applications [1]. The high-performance thermoplastic materials used for polymer gears, besides the weight factor, offer many advantages compared with conventional metal gears, such as the possibility to operate without lubrication, better noise properties, vibration damping, ease of production, and cost-effective manufacturing [2]. However, the use of polymer gears is still quite limited considering some shortcomings compared with metal gears, such as weaker mechanical properties, inferior thermal conductivity, lower operating temperatures, and a variety of failure modes that are induced not only by material properties but also with complex operating cycles defined by a wide scope of rotational speeds and loads, gear types, geometrical properties, lubrication conditions, and transmission configurations [3,4,5].

With the wide range of polymer gear applications, designers need to know many parameters that will most certainly vary for every different application [6,7,8]. Therefore, considering the limited data on existing polymer materials, a large number of the design parameters must be determined through experimental testing, which, to the greatest extent possible, needs to replicate the actual operating conditions [9,10]. Although this approach gives reliable results, these expensive and time-consuming tests are frequently used to identify the impact of an induced failure mechanism on the operational lifetime of polymer gears, which is not always an easy task, as several failure mechanisms may occur simultaneously [11,12]. In addition, knowledge of the sensitivity of a large number of design parameters and their progression on the service life of polymer gears is crucial for any polymer gear application, as the failure characteristics in different lifetime stages must be described reasonably well [13,14,15]. 

Surface wear is one of the major failure mechanisms in polymer gears, experienced at the interface of dry-running steel and polymer engagement. Although this standard engagement has a positive effect on the temperature properties of polymer gears, as the steel pinion, due to superior thermal conductivity, neglects most of the thermal effects on polymer gears, the softer, i.e., polymer gears, surface is prone to wear due to hardness dissimilarity between the surfaces in contact [16,17]. Particularly in dry-running conditions that extend the range of application opportunities in cases where lubrication is not allowed or hard to apply, the micro-asperities of the steel pinion directly penetrate through softer polymer material, resulting in an abrasive wear mechanism, as there is no lubrication film separating the surfaces. A direct loss of polymer material that leads to changes in contact topography also alters the contact pressure distribution, sliding conditions, and stiffness properties that accelerate the occurrence of other failure mechanisms and have a great impact on noise and vibration characteristics as the gear excitations are conditioned by the surface geometry [18,19]. These changes, dictated not only by the tooth profile degradation but also by the mechanics of gear contact, i.e., the combined motion of sliding and rolling, influence the behavior of the abrasive wear mechanism, which can be divided into three different phases. The beginning of the engagement, i.e., the running-in phase, distinguished by high wear rates in a short period, is followed by the phase of linear wear, where the transfer layer of polymer material removed by the shear mechanism has a positive effect on wear properties, slowing down the initial wear propagation and causing a low progressive wear rate over many operating cycles [20]. This phenomenon is also called third-body abrasion. The third phase, also referred to as the accelerated or severe wear phase, is a transient phase of non-stationary operational conditions that results in a rapid increase in wear rate, leading to failure [21,22]. 

Currently, the only available guidelines for the polymer gear design process that consider the abrasive wear mechanism are the VDI 2736 guidelines, which were proposed in 2014 and contain a calculation approach to determine the average linear wear of dry-running polymer gears [23,24,25,26]. Although these guidelines are very important for the current state of the polymer gear design process, they contain very limited data on wear coefficients for only a few polymer materials, which limits their applicability when it comes to new polymer gear design. However, even the existing data on wear coefficients result in major setbacks when it comes to gear design and wear lifetime predictions, as the available data are obtained from standard tribological testing, i.e., pin-on-disc experiments that are, in general, very different from real-scale gear testing [27,28]. As any realistic wear lifetime prediction and tribological contact simulation require a proper value of the wear coefficient for the specific application, researchers have established several methods that can be used for real-time wear coefficient assessment, with optical methods being the most commonly used for progressive tracking of tooth profile degradation after a specific number of operating cycles [29]. An experimentally obtained wear coefficient has a great impact on any wear process simulation flow, as it represents a large number of tribological, operational, and geometrical parameters, subjected to dynamic conditions and transient loads, that dictate the wear process of contacting surfaces [30].

When simulating the surface contact between two bodies, knowing the extent to which the surfaces are in contact, i.e., contact morphology is of crucial importance for many machine elements operating in dry, boundary, and mixed lubrication conditions [31,32]. A solution to any contact problem with corresponding changes due to wear, which provides useful information regarding the functioning of any machine element, can be obtained with the BEM method, as it only requires the contact interface between the boundary surfaces in contact [33]. Contrary to the finite element method (FEM), BEM utilization significantly reduces memory usage and improves computational efficiency at the same time, even with a large number of boundary elements [34]. For complex contact problems that require the solution of surface roughness and plasticity, even with today’s computational strength, FEM-based simulations with a fully adapted mesh become too demanding, leaving the BEM method the only viable option [35,36].

To estimate the impact of the wear mechanism in the contact of surfaces in relative sliding, Archard’s wear formulation is widely used in many simulation-based contact mechanics models for various engineering applications [37,38]. According to Archard’s wear formulation, the probability of a fracture occurrence at a micro-asperity level is expressed in the form of a wear coefficient [39,40]. The wear coefficient, which is usually determined experimentally, not only is related to probability but also encompasses deviations due to approximations and assumptions during the model development, such as different effects related to material properties, macroscopic shapes of bodies in contact, surface roughness, lubrication regime, lubrication type, and machining marks [41,42]. Discretization of Archard’s wear formation allows for a more complex analysis of contact problems with the derived Archard’s equation that is applied locally, i.e., to each element of the solution domain in terms of wear depth generated by contacting surfaces moving a small distance while carrying the subjected load [43,44]. Even without having the wear coefficient calibrated, contact mechanics simulation employing Archard’s wear formulation offers a great advantage, as it can be used to provide a qualitative view of the wear distribution [45,46]. This can be very useful in the design process of a certain machine element surface interface, where the applied model can be very helpful for understanding where the wear will occur or whether it will critically affect the performance of the component or not, therefore suggesting a change in a design or a solution for minimizing the impact of wear on the performance [47,48].

Unlike other contact problems, the uniqueness of the wear problem, reflected in wear-induced changes of an initial Hertzian geometry, requires a solution of a contact pressure distribution that is not known in analytical formulation even for the simplest configurations [49,50]. The only known closed-form solution can be obtained with Winkler’s surface model, which treats a specific point of a surface interface as a local function of pressure. With this approximation, the surface contact interface can be mapped with Winkler’s foundation model as a linear arrangement of independent and identical springs or bars separated by a sufficiently small discretization step [51,52]. Solutions to different simulation cases, considering different boundary conditions and contact mechanics model development, are obtained by calibrating Winkler’s modulus, i.e., the spring stiffness that needs to correspond to some quantity of the full elasticity solution, such as maximum pressure or the contact area, i.e., contact half-width [53,54]. 

The solution to the wear contact problem that involves complex three-dimensional (3D) bodies that operate under multi-cyclic dynamic conditions often requires a detailed compliance analysis with corresponding variations in load conditions, i.e., stress state [55,56]. This results in a modified surface geometry at each wear cycle that requires an update of the simulated contact interface [57,58]. Although the BEM method is less time-consuming compared with FEM, as the contact elements that are the only elements active in the wear simulation process have a significant impact on the stiffness matrix derivation, various problem-based analytical models that replicate the actual contact compliance can be implemented to accelerate the stiffness computation and therefore enhance the overall performance of the developed model [59,60]. 

The main objective of this research is to develop a contact mechanics model for surface wear prediction of parallel-axis polymer gears that will be validated through experimental testing. The influence of surface wear on the contact pressure distribution, sliding conditions, stiffness properties, and corresponding changes in normal load will also be thoroughly investigated. As the polymer gears reach a wear-steady state after the running-in period, the term of the limiting profile will be introduced.

## 2. Materials and Methods

### 2.1. Wear Formulation

#### 2.1.1. Computational Modeling of the Wear Depth

**Figure 1:**
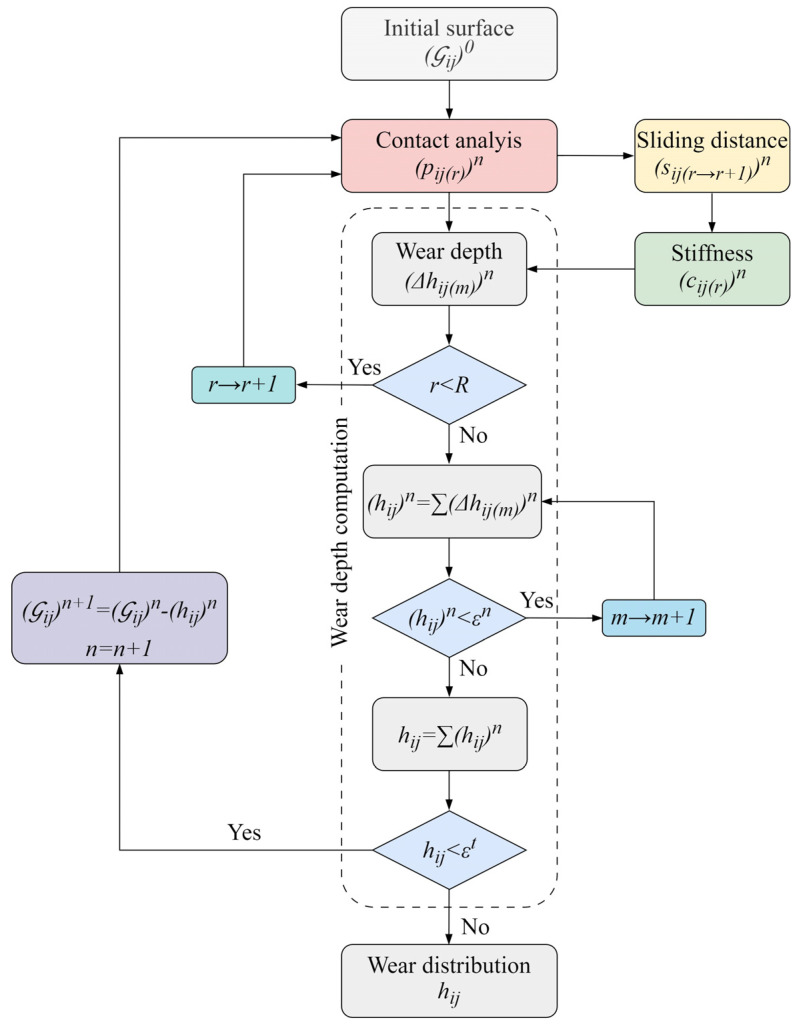
Computational flowchart of the contact mechanics model.

The developed contact mechanics model employs Archard’s wear formulation. With the assumption of constant hardnesses of contacting surfaces, the wear depth for a local point of a polymer gear tooth flank can be expressed with the following Equation (1):

(1) $$\frac{dh}{ds}=k_{w}\cdot p$$

where h is the wear depth, s is the sliding distance, $k_{w}$, is the dimensional wear coefficient, and p is the local contact pressure. Although the model can operate with an arbitrary value of the wear coefficient $k_{w}$, which can be used to easily estimate the sensitivity under various conditions and parameters, $k_{w}$, within the scope of this research, will be determined experimentally so it corresponds to polymer gear wear experiments that will be used for model validation [16]. A computational flowchart of the contact mechanics model, based on iterative numerical methodology, is shown in Figure 1. 

${\left(G_{ij}\right)}^{n}$ denotes the deviation of the arbitrary tooth flank surface point ij from the theoretical involute surface. The exponent n marks the number of geometry updates performed by the contact mechanics model. For n=0, the initial tooth flank surface, ${\left(G_{ij}\right)}^{0}$, equals the perfect involute surface with zero wear [22]. The next step in the surface wear prediction methodology is the computation of contact pressure distribution ${\left(p_{ij\left(r\right)}\right)}^{n}$ at each surface point ij, with the geometric data, ${\left(G_{ij}\right)}^{n}$, as an input. The separate deformable-body model for the computation of contact pressure distribution covers a complete wear cycle of tooth engagement at each rotational position r. The corresponding wear-induced changes in sliding conditions, ${\left(s_{ij(r\to r+1)}\right)}^{n}$, and stiffness properties, ${\left(c_{ij(r)}\right)}^{n}$, at each rotational position, r, are the next steps in the wear prediction methodology. As shown in Figure 1, the wear depth of an arbitrary node ij is summed up until the predefined value of $\varepsilon^{n}$, which will most certainly guarantee pressure update, is reached. This summation of wear depth, i.e., the iterative numerical procedure is then repeated until the termination criteria, i.e., the threshold value, $\varepsilon^{t}$, is reached. Depending on the problem being solved, the value of $\varepsilon^{t}$ can be represented as the maximum allowable wear depth or critical number of operating cycles that will, under certain load conditions, lead to a functional failure of polymer gear. After the threshold value, $\varepsilon^{t}$, is reached, the developed contact mechanics model outputs the wear distribution $h_{ij}$. 

#### 2.1.2. Geometric Description of Tooth Flank Surface

**Figure 2:**
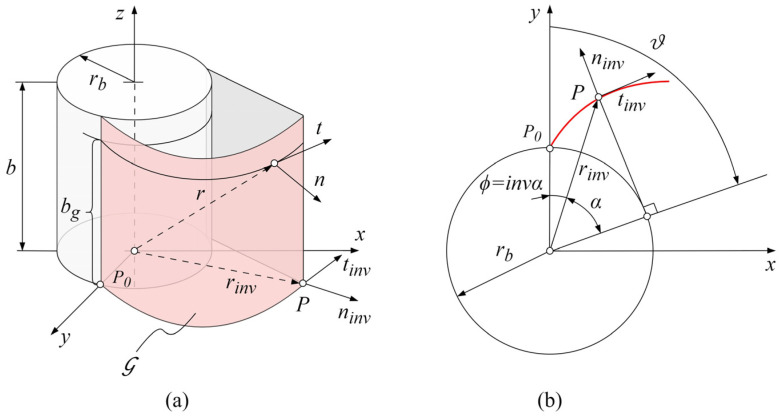
Tooth flank of a spur gear: (**a**) geometry; (**b**) tooth cross section.

The initial geometric data, ${\left(G_{ij}\right)}^{0}$, incorporated into the model can be described parametrically according to the tooth flank geometry [1]. Here, the focus will be put on the spur gears with involute geometry relevant to the wear analysis and simulation, as will be described later. If the coordinate frame origin is set to overlap with the center of the gear, with the y-axis crossing the starting point of the involute profile, $P_{0}$, at the base circle of the radius $r_{b}$, as shown in Figure 2a, each cross section of the tooth flank can be defined with the involute pressure angle α and the involute function ϕ or with the mutual variable parameter ϑ, which represents the involute roll angle, as shown in Figure 2b. 

The position vector $r_{inv}(\vartheta )$ of an arbitrary point P of an involute tooth profile shown in Figure 2b can be represented in matrix form, expressed by the following Equation (2):

(2) $$r_{inv}\left(\vartheta \right)=\left[\begin{array}{c} r_{b}(sin\vartheta -\vartheta cos\vartheta )\\ r_{b}(cos\vartheta +\vartheta sin\vartheta )\\ 0\\ 1 \end{array}\right]$$

with the involute roll angle ϑ, which needs to be defined between the boundary values that correspond to the start and end of the active profile, $\vartheta^{\left(s\right)}$ and $\vartheta^{\left(e\right)}$, respectively, as represented by the following Equation (3):

(3) $$\vartheta^{\left(s\right)}\leq \vartheta \leq \vartheta^{\left(e\right)}$$

The geometry of the tooth flank of a spur involute gear, G, can be represented as a locus of successive positions of the involute tooth profile, $r_{inv}(\vartheta )$, that moves in the z-axis direction. Accordingly, this motion can be described with the parameter of the tooth profile location $b_{g}$, as shown in Figure 2a, which yields the expression for the position vector of any point on the tooth flank of a spur involute gear, as presented in the following Equation (4):

(4) $$r\left(b_{g},\vartheta \right)=\left[\begin{array}{c} r_{b}(sin\vartheta -\vartheta cos\vartheta )\\ r_{b}(cos\vartheta +\vartheta sin\vartheta )\\ b_{g}\\ 1 \end{array}\right]$$

where the parameter of the tooth profile location $b_{g}$ needs to be defined within the gear width b, as expressed by the following Equation (5):

(5) $$0\leq b_{g}\leq b$$

It is important to emphasize that the developed contact mechanics model can use the geometric description of any tooth shape for wear prediction. For example, even the previously established geometric data can be easily adjusted for the wear problem of helical gears, just by introducing the helix angle for the rotation of the successive tooth profiles.

### 2.2. Contact Pressure Distribution

#### 2.2.1. Winkler’s Surface Model

**Figure 3:**
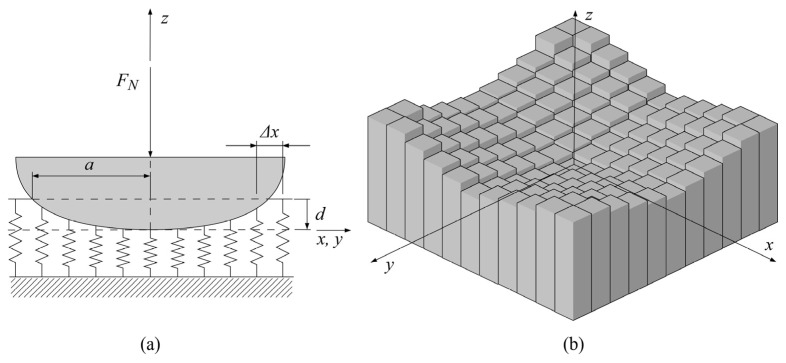
Winkler’s surface model: (**a**) schematic view; (**b**) 3D elastic foundation model.

As shown in Figure 1, the second step in the wear depth prediction is the computation of contact pressure distribution. The polymer gear geometric data ${\left(G_{ij}\right)}^{n}$ are an input to a separate model for the computation of contact pressure distribution, ${\left(p_{ij(r)}\right)}^{n}$, at different rotational positions, 0≤r≤R, of the meshing teeth, with the rotational increment sufficiently small to cover the complete wear cycle between the beginning of the engagement, r=0, and the end of the engagement, r=R. The contact pressure distribution computation methodology applied in this study is based on Winkler’s surface model [51,52]. According to this model, the displacement of an arbitrary surface point is a local function of pressure, with the surface interface being mapped with springs or bars equally separated by the distance ∆x, as shown in Figure 3a, or, if the contact problem requires, with the 3D elastic foundation model, as shown in Figure 3b. 

Figure 3a presents a schematic view of the surface interface with Winkler’s half-space where the deflection of points on the contact interface at any wear cycle is defined by the normal load $F_{N}$, contact half-width a, and indentation depth d. 

An elastic foundation model, i.e., Winkler’s hypothesis, considering wear-induced changes in the contact geometry interface, can be presented with the wear-dependent displacement field, as expressed by the following Equation (6):

(6) $$p(x,y,t)=K_{W}\cdot u(x,y,t)$$

where p(x,y,t) is the local pressure at the x,y location of the contact area, $K_{W}$ is Winkler’s modulus, and u(x,y,t) is the displacement field. As shown in Equation (6), p is the function of u, with both of them being time-varying quantities and $K_{W}$ being the proportionality coefficient only for the case of constant modulus. The wear-dependent displacement field can be described in terms of complementary variables of the contact interface, as expressed by the following Equation (7):

(7) $$u\left(x,y,t\right)=d\left(x,y,t\right)-f^{\left(0\right)}\left(x,y\right)-h(x,y,t)$$

where $f^{\left(0\right)}(x,y)$ is the initial gap function often referred to as the initial contact profile that approximates the contact interface as the 3D elastic continuum, i.e., the contact of a rigid indenter and elastic half-space.

#### 2.2.2. Winkler’s Modulus

**Figure 4:**
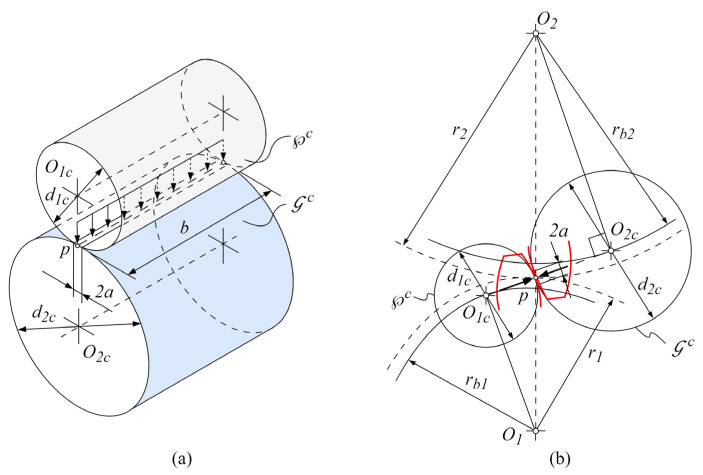
Instantaneous gear contact: (**a**) equivalent cylinders *℘^c^* and $G^{c}$ of pinion and gear, respectively; (**b**) derived contact at the pitch diameters $r_{1}$ and $r_{2}$ of pinion and gear, respectively.

Winkler’s modulus is usually a constant value chosen to agree with the solution of a non-conformal contact problem. Although some contact problems, due to complexity and the precision of solution, require the establishment of the time-varying modulus, i.e., adaptive Winkler’s modulus, the modulus within the developed contact mechanics model will be derived with the constant value corresponding to the peak Hertzian contact pressure [41,50]. The instantaneous contact between the meshing teeth at each rotational position, 0≤r≤R, can be considered analogous to a pair of equivalent cylinders with variable radiuses along the line of contact, as shown in Figure 4a,b.

As the width of the contact zone a at the rotational position r is much smaller than the radius of curvature and other geometric dimensions, the equivalent cylinder approach for the calculation of contact pressure distribution can be even simplified further by the contact of the equivalent cylinder and elastic half-space with the resultant radius and elastic property. Physically, the pressure distribution, fitting the contact area at the arbitrary rotational position r, yields the peak pressure, $P_{H}$, at the center of the contact area and the zero pressure at the edge of contact, as expressed by the following Equation (8):

(8) $$p\left(x,y\right)=p\left(x\right)=P_{H}\cdot \sqrt{1-\frac{x^{2}}{a^{2}}}$$

where $P_{H}$ is the maximum Hertzian pressure at x=0, i.e., the center of the contact. Accordingly, the derivation of Winkler’s modulus can be described with the following Equation (9):

(9) $$\left\{\begin{array}{c} x<a\left(x,0\right)\to p\left(x,0\right)=K_{W}\cdot \left[d\left(x,0\right)-f^{\left(0\right)}(x)\right]\\ x\geq a\left(x,0\right)\to p\left(x,t\right)=0 \end{array}\right.$$

where t is the time at which x=a(x,0), resulting in zero pressure. 

#### 2.2.3. Algorithm for the Computation of Contact Pressure Distribution 

Algorithm 1, i.e., the pseudo-code for the calculation of the contact pressure distribution at each rotational position r can be described in as follows.
**Algorithm 1** Contact pressure distribution**Initialize** *f^(0)^, d^(0)^, and p^(0)^*1   *Calculation of wear depth (hij)^n^*2   *Profile update f^(n)^* = *f^(n−1)^* + *(hij)^n^*3   *Calculation of new indentation depth d^(n)^*4   *Calculation of new contact pressure distribution p^(n)^*5   ***if** |f^(n+1)^ − f^(n)^| < tolerance go to step 6 else go to step 1*6   *Limiting profile p^(n)^ = const.****end***

At the edge of the contact, x=±a, the displacement of the elastic foundation equals zero, which yields the initial indentation depth expressed by the following Equation (10):

(10) $$u\left(x=\pm a\right)=0\to d=f^{\left(0\right)}(a)$$

By incorporating the wear coefficient, $k_{w}$, and sliding distance, ${s_{ij(r\to r+1)}}^{n}$, as the gears rotate from position r to position r+1, which will be discussed later, wear depth at any rotational position can be defined according to the following Equation (11):

(11) $${\left(h_{ij}\right)}^{n}=k_{w}\cdot p^{\left(n-1\right)}\cdot {\left(s_{ij(r\to r+1)}\right)}^{n}$$

The contact profile is then updated according to the following Equation (12):

(12) $$f^{\left(n\right)}\left(x\right)=f^{\left(n-1\right)}\left(x\right)+{\left(h_{ij}\right)}^{n}$$

Profile update at each wear cycle requires the knowledge of the new indentation depth, which can be derived using the equilibrium condition of the elastic foundation model, as expressed in the following Equation (13):

(13) $$F_{N}=\int_{-a}^{a}q_{z}(x)dx=2\cdot E*\cdot d^{\left(n\right)}\cdot a-2\cdot E*\int_{0}^{a}f^{\left(n\right)}\left(x\right)dx$$

where $q_{z}\left(x\right)$ is the linear force density and E* is the equivalent elastic property. With the new indentation depth, the field of displacement defined with Equation (7) gets updated, resulting in the new pressure distribution, as shown in Algorithm 1. The presented algorithm is applied to each rotational position, 0≤r≤R, which results, considering the discretization of the contact mechanics model, in a very complex model for the computation of contact pressure distribution [32]. 

Considering the problem of the gear meshing interface along with the fixed center distance, the surface of the tooth flank will eventually reach a limiting profile with a wear-steady state where the contact topography experiences quasi-linear changes with the constant distribution of contact pressure. The numerical solution to the limiting profile is shown in Algorithm 1, as the algorithm tracks the difference between consecutive contact profiles at each wear cycle. The algorithm is repeated until the difference between the contact profiles reaches a given tolerance, as expressed by the following Equation (14):

(14) $$\left|f^{\left(n+1\right)}-f^{\left(n\right)}\right|<tol\to p^{\left(n\right)}=const.$$

where the tolerance value tol, which is usually a very small quantity, is calibrated numerically to obtain the realistic and continuous contact pressure distribution, i.e., to best fit the analyzed contact problem.

### 2.3. Sliding Distance

**Figure 5:**
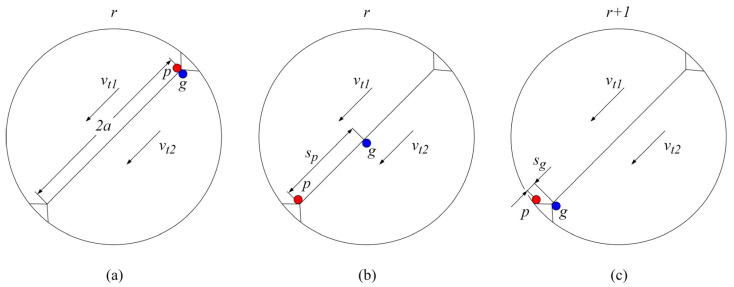
Sliding distance: (**a**) beginning of the engagement of points p and g, (**b**) the relative position of points p and g changes as the gears rotate, and (**c**) points p and g exit the contact zone.

The computation of the sliding distance, as the next step in the flowchart presented in Figure 1, for the arbitrary change in rotational position from r to r+1, is also founded on the equivalent cylinders approach, as presented in Figure 4. The sliding distance after n wear iterations, ${\left(s_{ij(r\to r+1)}\right)}^{n}$, can be thought of as the distance by which a point ij of a gear tooth surface performs the relative sliding movement concerning its mating point on the meshing surface, as shown in Figure 5.

Figure 5a presents the beginning of the engagement of mating points p and g of the pinion and gear, respectively, at the specific rotational position r. As the points p and g move with their respective tangential velocities, $v_{t1}$ and $v_{t2}$, they no longer overlap, as shown in Figure 5b. The rotation of gears from position r to r+1 results in points p and g moving the distance of contact width 2a, as shown in Figure 5c. Accordingly, the relative sliding distances $s_{p}$ and $s_{g}$, shown in Figure 5b,c, of the mating points p and g can be expressed with the following Equation (15) and Equation (16), respectively:

(15) $$s_{p}=2a\cdot \left(1-\frac{v_{t2}}{v_{t1}}\right)$$

(16) $$s_{g}=2a\cdot \left(1-\frac{v_{t1}}{v_{t2}}\right)$$

### 2.4. Meshing Stiffness

The crucial step for the development of the contact mechanics model is the computation of meshing stiffness, ${\left(c_{ij(r)}\right)}^{n}$, at each rotational position 0≤r≤R. As the normal load, $F_{N}$, is directly related to the meshing stiffness, the wear-induced changes in the tooth profile geometry, which will most certainly lead to compromised stiffness, also have a great influence on normal load distribution. The stiffness computational model, which uses gear geometric data ${\left(G_{ij}\right)}^{n}$ as an input, is based on Weber’s stiffness model. This model is founded on an analytical approach, and it is widely used for the computation of stiffness in gears as it is easy to apply compared with other stiffness computation models. 

#### 2.4.1. Weber’s Stiffness Model

**Figure 6:**
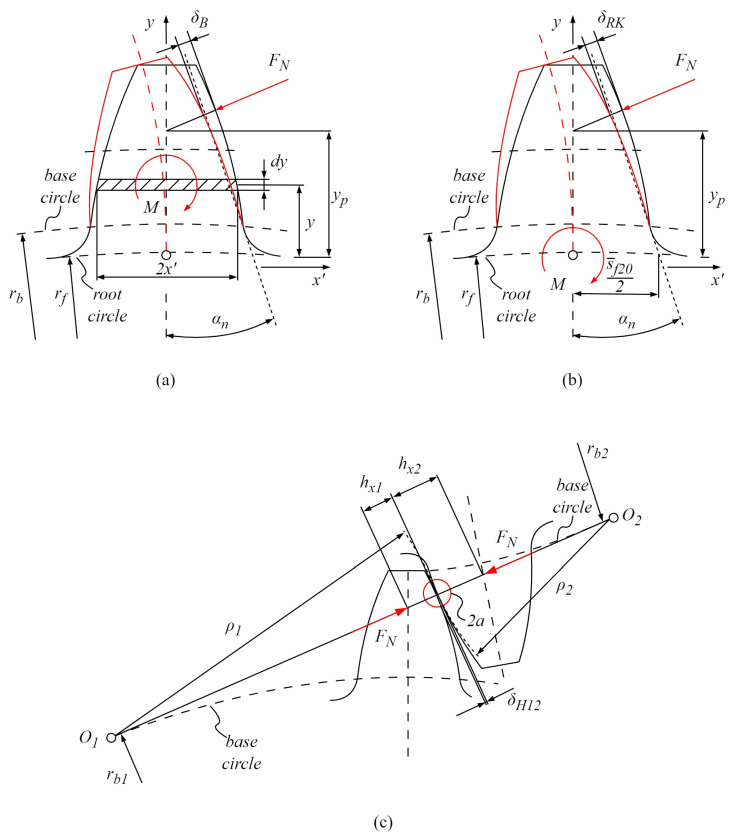
Tooth deformation according to Weber’s model: (**a**) bending deformation $\delta_{B}$, (**b**) gear body deformation $\delta_{RK}$, and (**c**) Hertzian contact deformation $\delta_{H}$.

According to Weber’s model, tooth stiffness, c, is composed of several components and can be expressed from the bending stiffness, gear body stiffness, and Hertzian contact stiffness, as represented in the following Equation (17):

(17) $$\frac{1}{c}=\frac{1}{c_{B}}+\frac{1}{c_{RK}}+\frac{1}{c_{H}}$$

where $c_{B}$ is the bending stiffness; $c_{RK}$ is the gear body, i.e., gear wheel stiffness; and $c_{H}$ is the Hertzian contact stiffness, with all of the stiffness components defined as a ratio of normal load $F_{N}$ and the appropriate deformation component, i.e., bending deformation $\delta_{B}$, gear body deformation $\delta_{RK}$, and Hertzian contact deformation $\delta_{H}$. Therefore, the solution to Weber’s stiffness model requires the computation of appropriate deformation components [55,56]. Figure 6a presents the bending deformation $\delta_{B}$. The bending deformation $\delta_{B}$, which is analogous to the Euler-Bernoulli cantilever beam supported at the gear root circle $r_{f}$ and bent by the moment M due to the action of the normal force $F_{N}$ at the current point of engagement, is expressed by the following Equation (18):

(18) $$\delta_{B}=\frac{F_{N}}{b}{cos}^{2}(\alpha_{n})\frac{1-\upsilon^{2}}{E}\left[12\int_{0}^{y_{P}}\frac{{\left(y_{P}-y\right)}^{2}}{{\left(2x^{\prime }\right)}^{3}}dy+\left(\frac{2.4}{1-\upsilon }+{\left(tan\alpha_{n}\right)}^{2}\right)\int_{0}^{y_{P}}\frac{dy}{2x^{\prime }}\right]$$

where $\alpha_{n}$ is the normal pressure angle; υ is Poisson’s ratio; E is the modulus of elasticity, i.e., the elastic property of the material; $y_{P}$ is the pitch point y-coordinate; y is the y-coordinate of the contact point, i.e., the rotational position r; and $x^{\prime }$ is the tooth half-width at the engagement point. Figure 6b presents the deformation of the gear body at the rooth circle $r_{f}$, caused by the bending moment M induced by the action of the normal force $F_{N}$. According to Weber’s model, the tooth is considered a rigid body, while the gear body is simplified as an elastic half-space, which leads to the additional tooth deflection $\delta_{RK}$ expressed by the following Equation (19):

(19) $$\delta_{RK}=\frac{F_{N}}{b}{cos}^{2}(\alpha_{n})\frac{1-\upsilon^{2}}{E}\left[\frac{18}{\pi }\cdot \frac{y_{P}^{2}}{{\bar{s}}_{f20}^{2}}+\frac{2-4\upsilon }{1-\upsilon }\cdot \frac{y_{P}}{{\bar{s}}_{f20}}+\frac{4.8}{\pi }\left(1+\frac{1-\nu }{2.4}{tan}^{2}(\alpha_{n})\right)\right]$$

where ${\bar{s}}_{f20}$ is the tooth width at the root circle. Hertzian contact deformation, shown in Figure 6c, results from the interference of the tooth flank surfaces with the radiuses of curvature, $\rho_{1}$ and $\rho_{2}$, at the arbitrary rotational position r. Based on the approximation of infinite half-spaces, the Hertzian contact deformation can be expressed by the following Equation (20):

(20) $$\delta_{H}=\frac{{2F}_{N}}{\pi b}\left\{\left(\frac{1-\upsilon_{p}^{2}}{E_{p}}\right)\left[ln\frac{2h_{xp}}{a}-\frac{\upsilon_{p}}{2\left(1-\upsilon_{p}\right)}\right]+\left(\frac{1-\upsilon_{g}^{2}}{E_{g}}\right)\left[ln\frac{2h_{xg}}{a}-\frac{\upsilon_{g}}{2\left(1-\upsilon_{g}\right)}\right]\right\}$$

where $h_{xp,g}$ is the distance between the contact point and the tooth axis of the pinion and gear, respectively.

The total deformation of the meshing teeth, i.e., the contact interface, can be expressed by the following Equation (21):

(21) $$\delta =\delta_{Bp}+\delta_{RKp}+\delta_{Bg}+\delta_{RKg}+\delta_{H}$$

Specific pair stiffness, i.e., stiffness at the single-tooth engagement period, which can be used for the derivation of the dynamic stiffness model, is expressed with the following Equation (22):

(22) $$c_{sp}=\frac{F_{N}}{\delta }=\frac{1}{\frac{1}{c_{Bp}}+\frac{1}{c_{RKp}}+\frac{1}{c_{Bg}}+\frac{1}{c_{RKg}}+\frac{1}{c_{H}}}$$

#### 2.4.2. Load-Sharing Factor

As there is a change in load conditions due to the rotation of gears, which can be divided into a single-tooth engagement period and a double-tooth engagement period for the engagements with a gear ratio between 1 and 2, it is important to account for the wear-induced changes of contact topography that result in alterations in normal load distribution [58]. Normal load at the arbitrary rotational position, r, can be defined based on the load-sharing factors, as expressed by the following Equation (23):

(23) $$F_{N}=\xi_{1}\cdot F_{N}+\xi_{2}\cdot F_{N}$$

where $\xi_{1,2}$ denotes the load-sharing factors that vary within certain limits and sum up to 1 at any rotational position 0≤r≤R. To describe the double-tooth engagement period, with the wear-induced alterations in normal load, these load-sharing factors can be defined concerning the meshing stiffnesses during the double-tooth engagement period, as expressed with the following Equations (24) and (25):

(24) $$\xi_{1}=\frac{c_{1}}{c_{1}+c_{2}}$$

(25) $$\xi_{2}=\frac{c_{2}}{c_{1}+c_{2}}$$

where $c_{1,2}$ denote the stiffnesses of gear pairs during the double-tooth engagement period. For the single-tooth engagement period, the normal load will equal the total meshing force, which is tangent to the gear base circle, as expressed by the following Equation (26):

(26) $$F_{N}=\frac{T_{d}}{r_{b}}$$

where $T_{d}$ is the torque and $r_{b}$ is the radius of the base circle.

#### 2.4.3. Algorithm for the Computation of Meshing Stiffness and Normal Load Distribution

The pseudo-code for the computation of meshing stiffness and normal load distribution at each rotational position r is presented with Algorithm 2.
**Algorithm 2** Meshing stiffness and load-sharing factors**Initialize** *($G_{\mathrm{ij}}$)^0^, (p_ij(r)_)^0^, and (s_ij(r__→__r+1)_)^0^*1   *Calculation of deformations (δ_Bp_)^n^, (δ_RKp_)^n^, (δ_Bg_)^n^, (δ_RKg_)^n^, and (δ_H_)^n^*2   *Calculation of stiffnesses (c_sp_)^n^, (c_1_)^n^, and (c_2_)^n^*3   *Calculation of load-sharing factors (ξ_1_)^n^ and (ξ_2_)^n^*4   *Calculation of normal load distribution F_N_ = (ξ_1_F_N_)^n^+(ξ_2_F_N_)^n^*5   ***if***
*n < stopping criteria update ($G_{\mathrm{ij}}$)^n^, (p_ij(r)_)^n^, and (s_ij(r__→__r+1)_)^n^ and go to step 1 **else** go to step 6*6   ***return***
*(c_sp_)^n^ and F_N_****end***


### 2.5. Computation of Wear Depth

As shown in the computational flowchart of the contact mechanics model, presented in Figure 1, if the wear depth accumulated after n simulated cycles is less than a predefined value of $\varepsilon^{n}$, wear depth is summed up for a certain number of cycles until it reaches $\varepsilon^{n}$. The value of the parameter $\varepsilon^{n}$, which is determined numerically, has to guarantee a pressure distribution update. Once the value of $\varepsilon^{n}$ is reached, after m wear updates, the surface geometry is updated, and the contact mechanics model computes the new contact pressure distribution, ${\left(p_{ij(r)}\right)}^{n}$, as shown in Figure 1, followed by the update of the sliding distance, ${\left(s_{ij(r\to r+1)}\right)}^{n}$, and meshing stiffness, ${\left(c_{ij(r)}\right)}^{n}$. This procedure is looped n times until the threshold value, $\varepsilon^{t}$, of the maximum wear depth is reached. The computed wear depth of an arbitrary surface node ij after n pressure updates, i.e., $m^{n}$ wear updates, is expressed by the following Equation (27):

(27) $${\left(h_{ij}\right)}^{n}=\sum_{m=1}^{m^{n}}{\left(∆h_{ij(m)}\right)}^{n}$$

Accordingly, the computed wear depth of an arbitrary surface node ij that reaches the threshold value $\varepsilon^{t}$ can be defined by the following Equation (28):

(28) $$h_{ij}=\sum_{n=1}^{n}{\left(h_{ij}\right)}^{n}$$

The total number of wear cycles, $m^{tot}$, resulting in the summation of wear, is represented by the following Equation (29):

(29) $$m^{tot}=\sum_{n=1}^{n}m^{n}$$

Finally, the wear depth after l number of wear cycles, $m^{n-1}<l<m^{n}$, can be described by the following Equation (30):

(30) $${\left(h_{ij}\right)}^{l}={\left(h_{ij}\right)}^{n-1}+\sum_{m=1}^{{l-m}^{n-1}}{\left(\Delta h_{ij(m)}\right)}^{n}$$

### 2.6. Development of Surface Model

**Figure 7:**
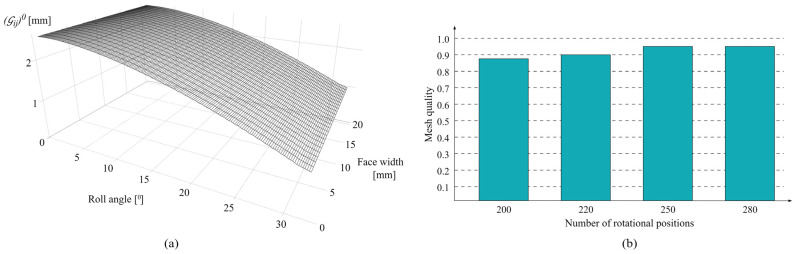
Contact mechanics model: (**a**) initial tooth flank surface; (**b**) mesh quality.

As the focus of the analyzed contact problem is tightly related to the surface interface and topography changes due to wear, the contact mechanics model formulation employs the BEM method [33]. This model covers the entire wear cycle of the active part of the tooth flank surface. As the contact half-width, a, at the arbitrary rotational position, r, is much smaller than other geometric quantities, the model requires a refined mesh for the proper solution of the moving contact problem. Figure 7a shows the contact mechanics model of the initial spur gear tooth flank with zero wear. Tooth flank geometry fully corresponds to the actual geometry of the gears that will be tested experimentally, with a face width of 20 mm and a profile line length of 6.67 mm.

For this specific gear geometry, which will be described in more detail in the next section, the gear rotation angle that corresponds to an entire wear cycle is nearly equal to 33°. The mesh at the profile direction, within the developed contact mechanics model, is formulated parametrically for the R=250 rotational positions that correspond to an angular increment of 0.128°. The necessary number of rotational positions is selected based on the recommendations of other researchers, so the contact half-width a is approximately 1/3 of the boundary element dimension in the profile direction. This number proved to be sufficient, as the h-refinement method was used to test the mesh density. The average reproducibility of the results compared with the higher number of rotational positions amounted to 95%, which corresponds to 0.95 mesh quality [2]. Mesh convergence results, regarding the analyzed contact parameters, are presented in Figure 7b. 

### 2.7. Wear Coefficient Assessment

#### 2.7.1. Data on Polymer Gears

The assessment of the wear coefficients was conducted through classic engagements that involve a steel gear and a polymer pinion, with a gear ratio of 1. Polymer gears, used in the experimental investigation, were made out of POM (DuPont, Neu-Isenburg, Germany) and PVDF (Arkema, Colombes, France). The basic geometric parameters of polymer gears are presented in Table 1. 

The geometric data presented in Table 1 correspond to the geometry of polymer gears with the same module in real-life applications, which are commonly used in various pump and conveyor systems. The POM material properties are shown in Table 2, while Table 3 presents the material properties of the PVDF material.

Considering the specified gear geometry and the operating conditions in real-life applications, polymer gears are experimentally tested on torque levels of 4 Nm, 5 Nm, and 6 Nm at a rotational speed of 1000 rpm. 

#### 2.7.2. Experimental Setup

**Figure 8:**
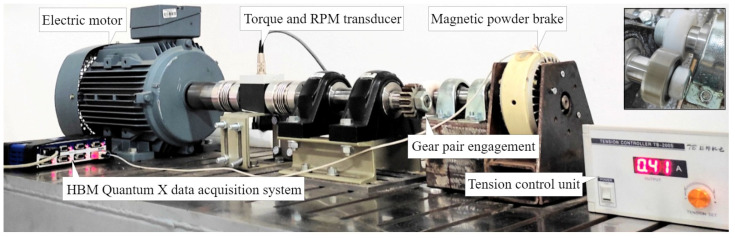
Custom-made test rig for polymer gears.

Testing of the polymer gears is conducted on a custom-made test rig, presented in Figure 8. The test rig, which is based on an open-loop configuration, obtains power through an electric motor (Marathon Electric HJA-IE2 132 M, Regal Rexnord Corporation, Wausau, WI, USA) controlled by the frequency regulator (EN600-4 T 0075G/110P, Shenzhen Encom Electric Technologies CO, Shenzen, China). The torque transducer (HBM T20WN T153040, Hottinger Baldwin Messtechnik GmbH, Darmstadt, Germany) is coupled to the Quantum X system for the acquisition of data on torque and rotational speed. The load is simulated with a magnetic powder brake (FZ-12-K TB-200S, Yun Duan, Taiwan, China) connected to the tension control unit used for the precise adjustment of the torque level. The test rig allows for the experimental testing of numerous gear geometries as the center distance can be adjusted by positioning the driven shaft in the horizontal direction [5].

#### 2.7.3. Wear Analysis 

**Figure 9:**
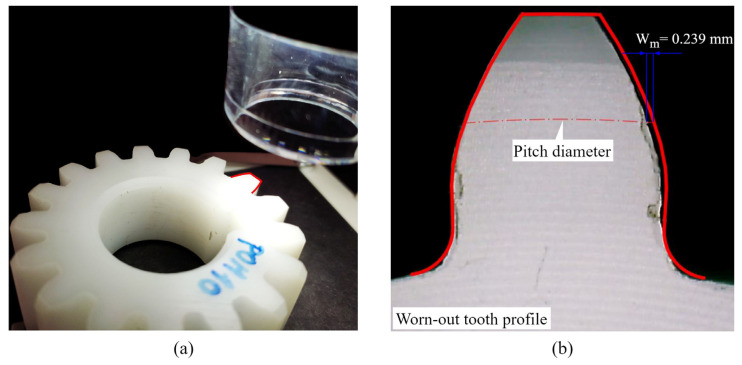
Wear coefficient assessment: (**a**) inspecting the side view of the worn-out tooth profile; (**b**) measuring the average linear wear.

The wear coefficients necessary for the formulation of the contact mechanics model are obtained by the wear model presented in the VDI 2736 guidelines for polymer gear design [24]. The wear coefficients can be determined by using a digital microscope (NB-MIKR-500), i.e., by employing the optical methods to compare the tooth profile before and after the test, as shown in Figure 9a. The maximum perpendicular distance between the theoretical and worn-out tooth profile represents the average value of linear wear $W_{m}$ used for the determination of the wear coefficient [3,5].

The worn-out tooth profiles are inspected at a 22-fold magnification with the averaged linear wear $W_{m}$ detected around the pitch diameter zone, as shown in Figure 9b. With the known value of $W_{m}$, the wear coefficient is calculated by the VDI 2736 abrasive wear model presented in the following Equation (31):

(31) $$k_{w}=\frac{W_{m}\cdot b_{w}\cdot z\cdot l_{FL}}{2\cdot \pi \cdot T_{d}\cdot N_{L}\cdot H_{V}}$$

where $b_{w}$ is the common face width, z is the number of teeth, $l_{FL}$ is the length of the profile line, $N_{L}$ is the number of load cycles, and $H_{V}$ is the degree of tooth loss. The data on the evaluated values of wear coefficients for POM and PVDF gears, which are handled as an input to the contact mechanics model, is presented in Table 4.

Considering that this study is focused on the development of the model for wear prediction, it is important to emphasize that, in this section, many great details have been omitted and the wear treatment is only outlined. For detailed data on experimental testing and wear coefficient assessment, the readers will be referred to our previous research [5].

## 3. Results

Considering the numerous contact parameters that can be obtained with the developed model at an arbitrary number of simulated load cycles, as well as the input data on wear coefficients determined for two polymer materials at three different load levels, only a limited amount of data will be presented in this section. The case study with a load level of 5 Nm and corresponding wear coefficients of 9.77 × 10^−6^ mm^3^/(Nm) and 10.98 × 10^−6^, for POM and PVDF gears, respectively, will be considered for a comparative analysis. 

### 3.1. Contact Mechanics Model Predictions for POM Gears

**Figure 10:**
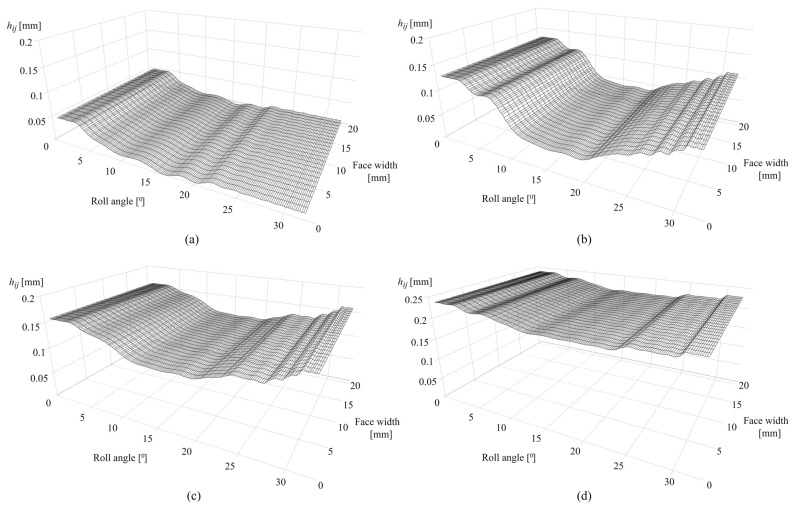
Wear distribution of POM gears: (**a**) 0.25 × 10^6^ cycles, (**b**) 1 × 10^6^ cycles, (**c**) 2.5 × 10^6^ cycles, and (**d**) 4.5 × 10^6^ cycles.

Figure 10a presents the wear distribution after 0.25 × 10^6^ cycles. The maximum wear depth, which amounts to 0.0506 mm, is located in the dedendum area of the tooth flank surface, near the start of the active profile for roll angles of less than 3°. The wear depth is negligible at the pitch line with a corresponding roll angle of 16.5°. 

As shown in Figure 10b, the maximum wear depth after 1 × 10^6^ cycles, which amounts to 0.129 mm, is located for roll angles of less than 3°. Meanwhile, the rest of the active profile experiences a further increase in wear, with a significant wear depth of 0.11 mm for roll angles of greater than 31°. The wear distribution after 2.5 × 10^6^ is presented in Figure 10c. The maximum values of wear depth with a value of 0.151 mm are located at the dedendum and addendum regions, while the rest of the active profile experiences a significant increase in wear. Figure 10d shows the wear distribution along the active tooth profile after 4.5 × 10^6^ cycles with approximately uniform wear depth distribution. 

**Figure 11:**
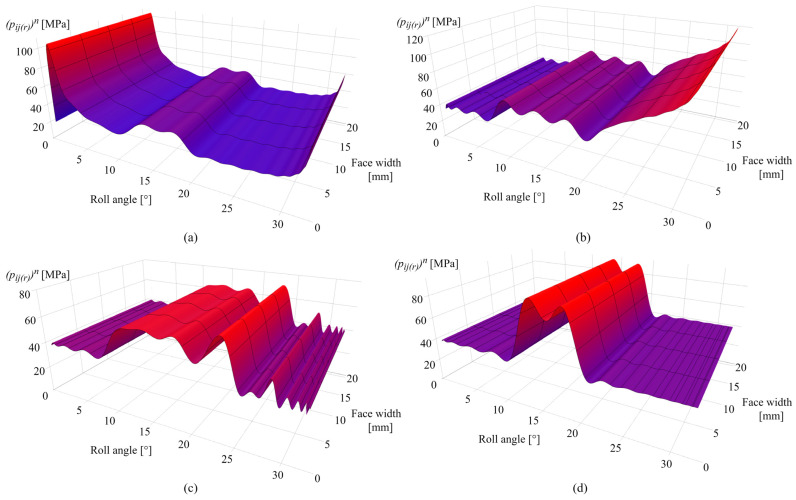
Contact pressure distribution of POM gears: (**a**) 0.25 × 10^6^ cycles, (**b**) 1 × 10^6^ cycles, (**c**) 2.5 × 10^6^ cycles, and (**d**) 4.5 × 10^6^ cycles.

Figure 11a presents the contact pressure distribution after 0.25 × 10^6^ cycles. As shown in Figure 11a, in the initial phases of engagement, the Hertzian pressure distribution is altered due to the wear mechanism, resulting in a peak pressure of 104 MPa at the zone of the maximum wear depth that corresponds to a roll angle of 2°. Figure 11b shows the contact pressure distribution after 1 × 10^6^ cycles. Notably, for roll angles of less than 6°, the limiting profile is reached with an approximately constant pressure value of 40 MPa.

As obvious from the previous contact pressure distribution images, the increase in contact pressure follows the corresponding changes in wear distribution. Figure 11c presents the contact pressure distribution after 2.5 × 10^6^ cycles with a maximum contact pressure of 70 MPa for a roll angle of 23°. Notable alterations in contact pressures, for roll angles of greater than 26°, result from the changes in sliding conditions and increase in wear intensity at the specific rotational positions. Figure 11d shows the contact pressure distribution after 4.5 × 10^6^ cycles with a maximum contact pressure of 90 MPa located around the pitch line zone (roll angles of 12° and 18°).

**Figure 12:**
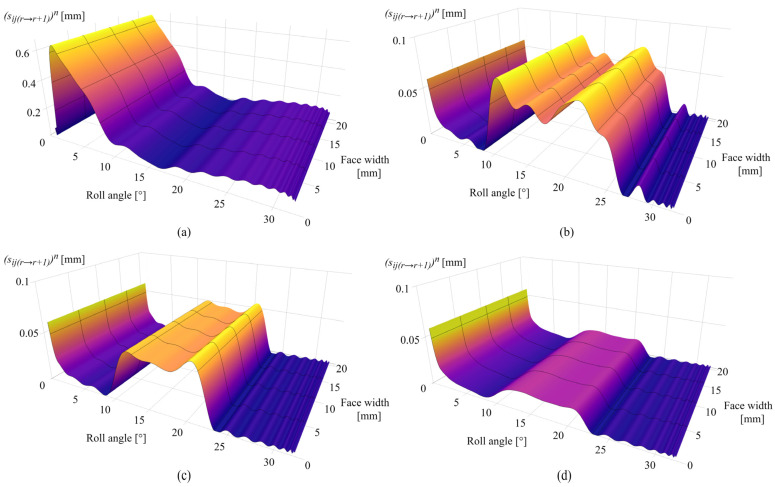
Sliding conditions of POM gears: (**a**) 0.25 × 10^6^ cycles, (**b**) 1 × 10^6^ cycles, (**c**) 2.5 × 10^6^ cycles, and (**d**) 4.5 × 10^6^ cycles.

Figure 12a presents the sliding conditions after 0.25 × 10^6^ cycles with a maximum sliding distance of 0.62 mm in the dedendum area at a roll angle of 2° and the negligible sliding at the pitch line, i.e., a roll angle of 16.5°. As the sliding conditions follow the changes in contact pressure distribution and wear distribution, the maximum sliding distance after 1 × 10^6^ cycles is located around the pitch line zone at roll angles of 10° and 21° with a value of 0.079 mm, as shown in Figure 12b. The decrease in the sliding conditions at the start of the active profile fully corresponds to the approximately constant pressure distribution shown in Figure 11b, i.e., the limiting profile. Figure 12c presents the sliding conditions after 2.5 × 10^6^ cycles. The maximum sliding distance, corresponding to changes in wear intensity according to Figure 10c, is located at a roll angle of 20° and amounts to 0.06 mm. The changes in sliding distance after 4.5 × 10^6^ cycles are presented in Figure 12d with maximum values of 0.055 mm at the start of the active profile and 0.02 mm at a roll angle of 21°. A spike in sliding conditions at the start of the active profile, notable in Figure 12b–d, is also in agreement with the gear theory, as the beginning of tooth engagement results in the greatest sliding at the root area.

**Figure 13:**
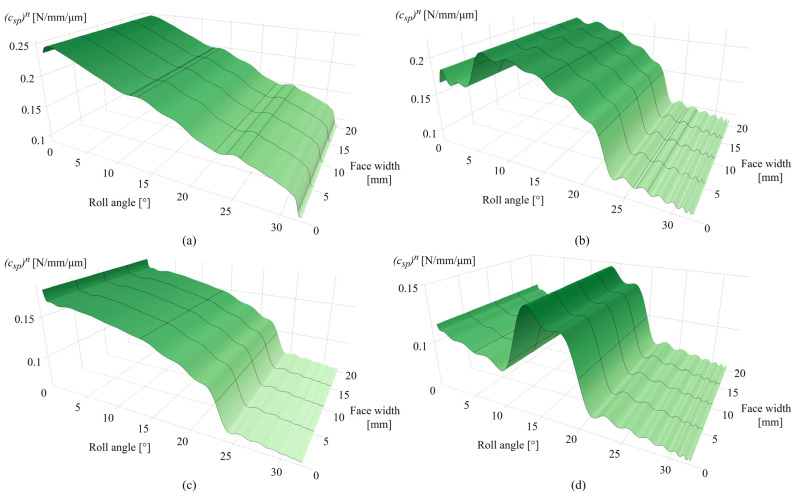
Specific pair stiffness: (**a**) 0.25 × 10^6^ cycles, (**b**) 1 × 10^6^ cycles, (**c**) 2.5 × 10^6^ cycles, and (**d**) 4.5 × 10^6^ cycles.

Considering many stiffness components that contribute to the overall meshing stiffness, as well as the corresponding tooth deformation components, only limited data on overall meshing properties will be presented. To easily encompass these wear-induced changes in meshing stiffness, the contact mechanics model is adjusted to output the specific pair stiffness with a custom y-axis scale. Figure 13a presents the specific pair stiffness after 0.25 × 10^6^ cycles with a maximum value of 0.247 N/mm/µm at the tooth root area. Figure 13b shows the changes in specific pair stiffness after 1 × 10^6^ cycles with a maximum of 0.22 N/mm/µm at a roll angle of 6 ° and the decrease in meshing stiffness at the dedendum and addendum areas due to wear. For the 2.5 × 10^6^ cycles, presented in Figure 13c, there is a further decrease in meshing stiffness along the active tooth profile. Figure 13d presents the specific pair stiffness after 4.5 × 10^6^ cycles, with the compromised stiffness at the dedendum and addendum areas and a maximum specific pair stiffness of 0.146 N/mm/µm at a roll angle of 12°. The stiffness alterations notable in Figure 13b–d are mostly affected by the wear-induced changes in Hertzian contact stiffness, as the wear-induced changes in the ratio of tooth thickness and contact half-width, expressed by Equation (20), lead to alterations in local contact compliance at a specific rotational position r. The stiffness values obtained from Weber’s computational model, considering the dynamic stiffness properties regarding the single- and double-tooth engagement periods, are used for the determination of load-sharing factors and corresponding distributions of normal load.

**Figure 14:**
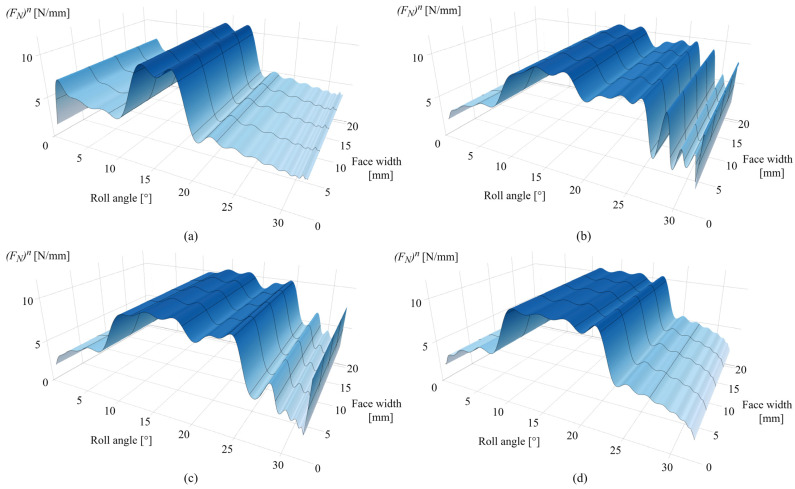
Normal load: (**a**) 0.25 × 10^6^ cycles, (**b**) 1 × 10^6^ cycles, (**c**) 2.5 × 10^6^ cycles, and (**d**) 4.5 × 10^6^ cycles.

Figure 14a presents the normal load distribution after 0.25 × 10^6^ cycles. As the initial wear occurs at the tooth root area, a slight increase in normal load is observed at a roll angle of 2°, with the rest of the active profile experiencing an approximately initial state of normal load distribution, with distinguished differences between the single- and double-tooth engagement periods. The corresponding changes of normal load distribution after 1 × 10^6^ cycles are presented in Figure 14b. Figure 14c,d present the normal load distributions after 2.5 × 10^6^ and 4.5 × 10^6^ cycles, respectively. As notable from the normal load distribution images, the wear-induced alterations in normal load correspond to the changes in meshing stiffness, with maximum values of 10.8 N/mm that correspond to the single-tooth engagement period.

### 3.2. Contact Mechanics Model Predictions for PVDF Gears

**Figure 15:**
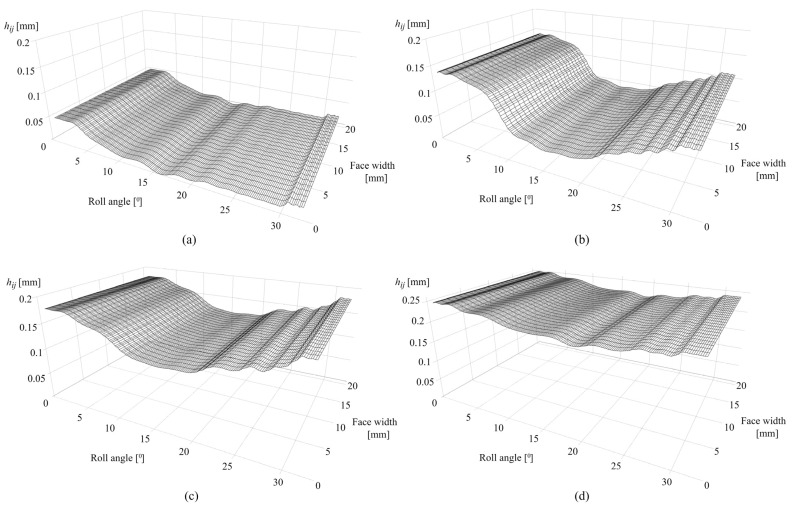
Wear distribution of PVDF gears: (**a**) 0.25 × 10^6^ cycles, (**b**) 1 × 10^6^ cycles, (**c**) 2.5 × 10^6^ cycles, and (**d**) 4.5 × 10^6^ cycles.

Figure 15a presents the wear distribution of PVDF gears after 0.25 × 10^6^ cycles with a maximum wear depth of 0.051 mm at a roll angle of 3°, i.e., dedendum area and negligible wear at the pitch line (roll angle of 16.5°). Compared with POM gears, there is a slight increase in wear along the active profile due to a higher value of wear coefficient. Figure 15b,c present the wear distributions after 1 × 10^6^ and 2.5 × 10^6^ cycles with maximum wear depths of 0.141 mm (roll angle of 2.5°) and 0.18 mm (roll angles of 2° and 31°), respectively. Figure 15d presents the wear distribution after 4.5 × 10^6^ cycles.

**Figure 16:**
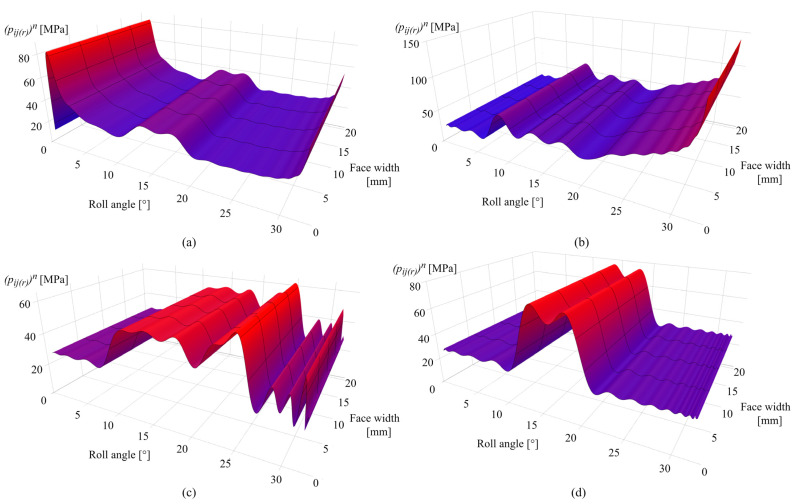
Contact pressure distribution of PVDF gears: (**a**) 0.25 × 10^6^ cycles, (**b**) 1 × 10^6^ cycles, (**c**) 2.5 × 10^6^ cycles, and (**d**) 4.5 × 10^6^ cycles.

Figure 16a presents the contact pressure distribution after 0.25 × 10^6^ cycles with a maximum pressure of 82 MPa at a rotational angle of 2°. As shown in Figure 16b, the limiting profile with an approximately constant pressure value of 25 MPa is reached for angles of less than 6°. The increase in contact pressure is notable for roll angles greater than 25° with a maximum value at the end of the active profile (roll angle of 33°), which amounts to 140 MPa. Figure 16c shows an increase in contact pressure distribution at the pitch line zone as well as for roll angles between 21° and 25°, which fully corresponds to the increase in wear at the same areas, as presented in Figure 15c. At a larger number of load cycles, contact with the most intense wear is shifted to the middle of the active profile, as presented in Figure 16d for the 4 × 10^6^ simulated load cycles, with a maximum pressure of 72 MPa at roll angles of 12° and 18°. Meanwhile, the rest of the active profile reached a wear-steady state, i.e., limiting profile.

**Figure 17:**
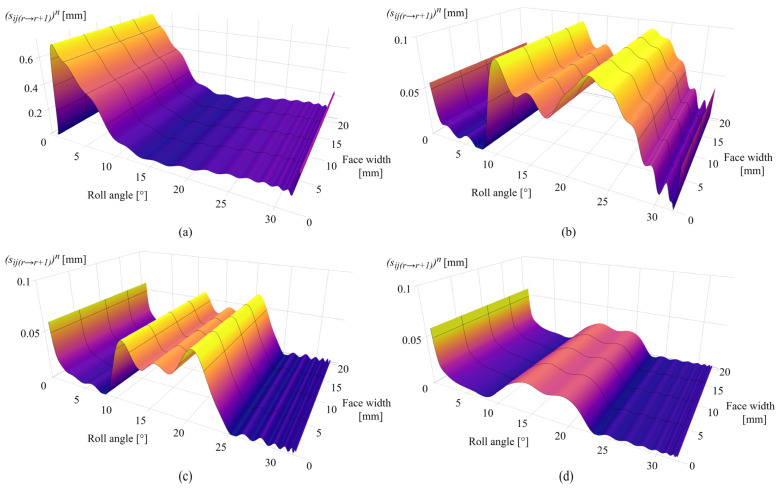
Sliding conditions of PVDF gears: (**a**) 0.25 × 10^6^ cycles, (**b**) 1 × 10^6^ cycles, (**c**) 2.5 × 10^6^ cycles, and (**d**) 4.5 × 10^6^ cycles.

Figure 17a presents the sliding conditions of PVDF gears after 0.25 × 10^6^ cycles. The maximum sliding distance, as expected in the early phases of engagement, is located at the tooth root area with a value of 0.68 mm at a roll angle of 2°. The increase in the sliding distance at the end of the active profile (roll angle of 33°) results from a local pressure increase at the same zone, as notable in Figure 16a. Figure 17b shows the sliding conditions after 1 × 10^6^ cycles with maximum values of sliding distances of 0.09 mm and 0.092 mm for roll angles of 10° and 23°, respectively. The sliding conditions after 2.5 × 10^6^ cycles are shown in Figure 17c with a maximum sliding distance of 0.07 mm at a roll angle of 20°. A further decrease in sliding conditions after 4.5 × 10^6^ load cycles is presented in Figure 17d. Images of the contact pressure distributions and sliding conditions, for both POM and PVDF gears at the different numbers of simulated load cycles, as presented, proved to be very useful, as it is easy to comprehend the behavior of these parameters concerning the wear distribution in different phases of engagement. Even in the images of sliding distances, it is possible to recognize the occurrence of the wear-steady state, i.e., reaching the limiting profile, as the sliding distances downgrade to approximately constant values at the same zones of constant pressure distribution.

**Figure 18:**
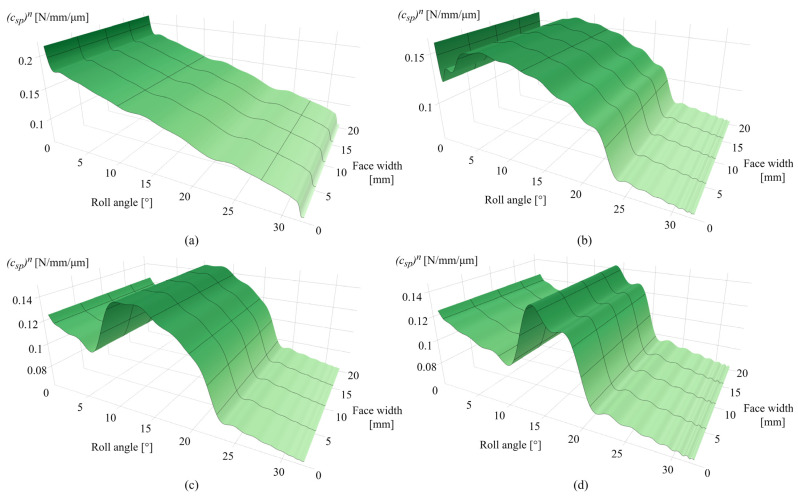
Specific pair stiffness: (**a**) 0.25 × 10^6^ cycles, (**b**) 1 × 10^6^ cycles, (**c**) 2.5 × 10^6^ cycles, and (**d**) 4.5 × 10^6^ cycles.

Figure 18a shows the specific pair stiffness after 0.25 × 10^6^ cycles, with a slight decrease in stiffness for a roll angle of 2° due to intensive wear at the root area in the early stages of engagement. The maximum value of specific pair stiffness amounts to 0.22 N/mm/µm at a roll angle of 0°, i.e., the start of the active profile. If compared with POM gears, meshing stiffness is lower due to the mechanical properties of the PVDF material. The corresponding decrease in specific pair stiffness for the 1 × 10^6^ and 2.5 × 10^6^ cycles corresponds to the zones of the most intensive wear, i.e., the dedendum and addendum area, as shown in Figure 18b,c, respectively. The maximum stiffness value, as shown in Figure 18b, amounts to 0.153 N/mm/µm at roll angles of 0° and 6°. For the 2.5 × 10^6^ load cycles, further propagation of the wear mechanism decreases the meshing stiffness for roll angles of less than 7°, as shown in Figure 18c. The maximum stiffness value is located at a roll angle of 12.5° and has a value of 0.143 N/mm/µm. Figure 18d presents the specific pair stiffness after 4.5 × 10^6^ cycles with a maximum value of 0.141 N/mm/µm for a roll angle of 12.5°.

**Figure 19:**
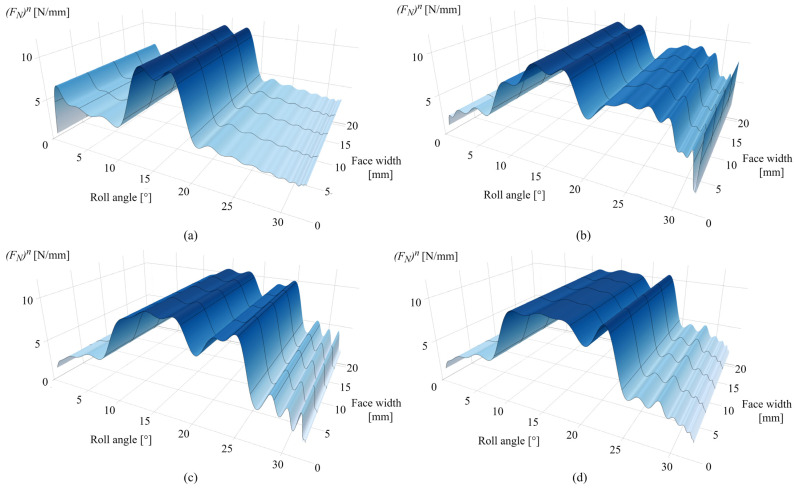
Normal load: (**a**) 0.25 × 10^6^ cycles, (**b**) 1 × 10^6^ cycles, (**c**) 2.5 × 10^6^ cycles, and (**d**) 4.5 × 10^6^ cycles.

The corresponding changes in normal load distribution after 0.25 × 10^6^ cycles are presented in Figure 19a. The increase in the normal load at the 2° roll angle corresponds to the spike of contact pressure at the same zone. Alterations in the normal load distribution after 1 × 10^6^ and 2.5 × 10^6^ cycles are presented in Figure 19b,c, respectively. Figure 19d shows the normal load distribution after 4.5 × 10^6^ cycles. As shown in these images, the normal load is shifted from an initial stage of distinguished single- and double-tooth engagement to an altered distribution that corresponds to the wear-induced changes in contact topography at a different number of load cycles.

### 3.3. Model Validation

**Figure 20:**
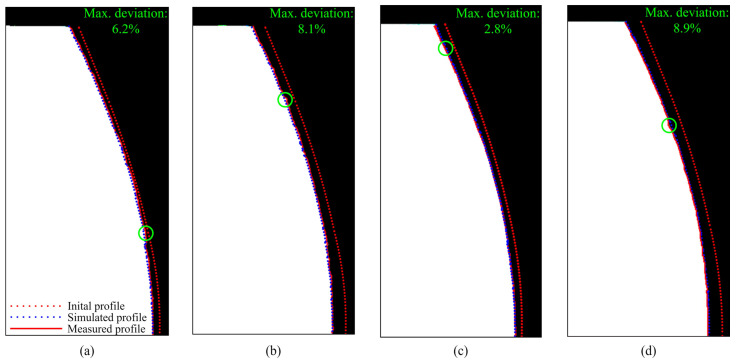
Comparison of the actual and simulated tooth profiles: (**a**) POM gears at a torque level of 4 Nm after 2 × 10^6^ cycles, (**b**) POM gears at a torque level of 5 Nm after 3.7 × 10^6^ cycles, (**c**) PVDF gears at a torque level of 4 Nm after 4 × 10^6^ cycles, and (**d**) PVDF gears at a torque level of 6 Nm after 2.5 × 10^6^ cycles.

**Figure 21:**
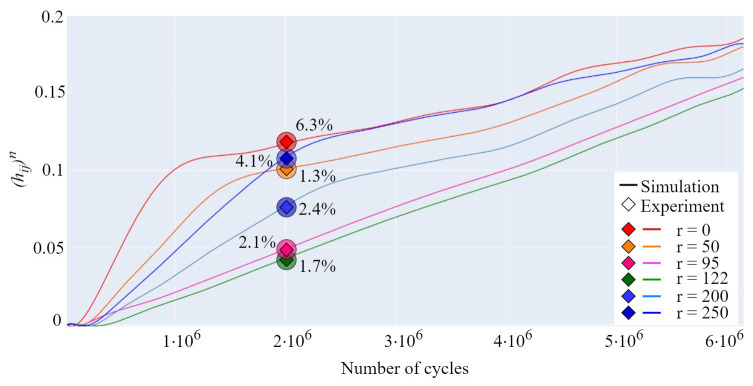
Wear curves of POM gears at a torque level of 4 Nm with the deviations between simulated and measured wear depths after 2 × 10^6^ cycles.

The developed contact mechanics model is validated by performing a series of experimental tests with the undefined stopping criteria; i.e., the testing is terminated after an arbitrary number of load cycles. Worn-out gear geometry is then compared with the simulated profiles obtained with the model. The measurement of the worn-out tooth profiles is performed with the digital microscope. The properties of the digital microscope allow for the creation of edge-recognition images of high accuracy (10 µm), which are imported into PortableCapture Plus 3.1 software for precise geometry measurement. Figure 20a presents the difference between the actual and simulated profiles of POM gears at a torque level of 4 Nm after 2 × 10^6^ cycles. The maximum deviation between the profiles, as shown in Figure 20a, amounts to 6.2%. As presented in Figure 20b, the maximum deviation between the actual and simulated profiles of POM gears at a torque level of 5 Nm and 3.7 × 10^6^ load cycles amounts to 8.1%. Figure 20c presents the difference between the actual and simulated profiles of PVDF gears at a torque level of 4 Nm after 4 × 10^6^ cycles, with a maximum deviation of 2.8% located at the addendum area. For PVDF gears at a torque level of 6 Nm and 2.5 × 10^6^ load cycles, a maximum deviation of 8.9% is established, as shown in Figure 20d. As shown in Figure 21, the maximum deviations, which are denoted with green circles, are located at different areas of the tooth profile. For a better insight into the deviations of simulated worn-out profiles, the developed contact mechanics model offers the possibility to output the wear curves for any rotational position r. As a demonstrative example, Figure 21 presents the wear curves for the specific rotational positions of POM gears at a torque level of 4 Nm. The deviations of the wear curves regarding the experimental data, denoted with circles, are presented for 2 × 10^6^. Even though the model can output the wear curves for any rotational position, the comparison has been made for several curves only due to a large amount of data. The rotational positions r=0 and r=250 denote the start and the end of the active profile, respectively, while the positions r=95 and r=122 present the points in the vicinity of the pitch line. As shown in Figure 21, the different phases of wear intensity at any rotational position can be observed based on the slope of wear curves. 

**Figure 22:**
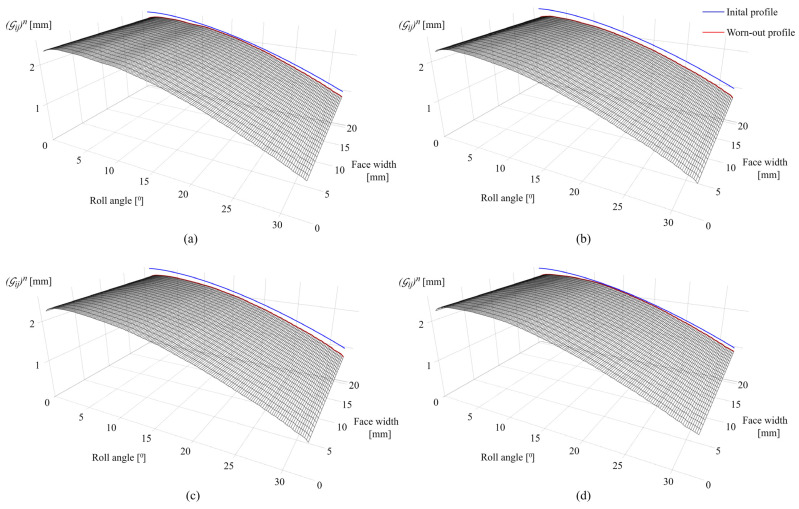
Worn-out tooth surfaces: (**a**) POM gears at a torque level of 4 Nm after 2 × 10^6^ cycles, (**b**) POM gears at a torque level of 5 Nm after 3.7 × 10^6^ cycles, (**c**) PVDF gears at a torque level of 4 Nm after 4 × 10^6^ cycles, and (**d**) PVDF gears at a torque level of 6 Nm after 2.5 × 10^6^ cycles.

Figure 22a,b present the worn-out tooth surfaces of POM gears at torque levels of 4 Nm and 5 Nm after 2 × 10^6^ and 3.7 × 10^6^, respectively. The worn-out tooth surfaces of PVDF gears at torque levels of 5 Nm and 6 Nm after 4 × 10^6^ and 2.5 × 10^6^ are presented in Figure 22c,d, respectively. 

## 4. Discussion

As shown in this study, the developed contact mechanics model for the wear prediction of parallel-axis polymer gears presents a complex and multidisciplinary model that requires extensive knowledge of various mechanical and tribological parameters, as well as the description of the various geometric shapes and interfaces of surfaces in contact, and the corresponding changes that occur due to wear. Even though the presented methodology applies to parallel-axis gear pairs, the extension of the presented contact mechanics model is possible for arbitrary gear shapes, as the model can easily incorporate any geometric description, as well as the more complex formulations of sliding conditions that are expected for the other tooth geometry [1,30]. 

Archard’s wear model, which was employed in the wear depth computation, proved to be sufficient for the description of wear distribution at different numbers of load cycles. Even though this relatively simple wear formulation can be used without the knowledge of the wear coefficient, $k_{w}$, to qualitatively describe the wear distribution for a specific contact problem, the expected value of the wear coefficient in real-life applications is necessary for realistic wear predictions [16,27]. More advanced wear formulations that require data on surface roughness parameters and thermal wear behavior can also be easily implemented into the presented methodology.

Winkler’s surface model, employed within the developed contact mechanics model, presents the basic elastic foundation model that treats the contact at any rotational position as non-adhesive; i.e., it does not take into account the lateral effects at the contact zone. Accordingly, the extension to this model can also be applied by determining the coefficients of friction at the engagement zone using the Hachmann–Strickle temperature model [24]. However, an extensive thermal investigation would be required. Winkler’s moduli within the scope of this study are determined from the initial contact conditions that correspond to the Hertzian solution and amount to 4007 MPa/mm and 2880 MPa/mm, for POM and PVDF gears, respectively. It is clear from the conducted research, that, after a specific number of load cycles, the contact pressure distribution becomes invariable to the values of Winkler’s moduli with the changes in sliding conditions, i.e., the transformation of the initial Hertzian contact to a conformal contact. The latter can be solved by employing an adaptive Winkler’s modulus with time-varying values [50]. 

As the polymer gears, due to the specificity of the contact problem, reach a wear-steady state, i.e., a limiting profile with approximately constant pressure distribution and sliding conditions, it is necessary to track the wear-induced changes at the contact topography, i.e., the changes in contact profile at each rotational position, r. The tolerance value, tol, between two consecutive contact profiles has to result in realistic pressure values that agree with the previous contact pressure distribution and needs to be determined numerically by the trial and error method [32]. The tolerance value, tol, employed in the presented contact mechanics model amounts to 1 × 10^−11^ mm.

The predefined value of $\varepsilon^{n}$, which needs to guarantee a pressure update, is also determined numerically. The developed model implements a value of 10 µm, which also corresponds to the accuracy of the digital microscope, i.e., the obtained edge-recognition images of the worn-out tooth profiles. Even though the model can work with an arbitrarily smaller value of $\varepsilon^{n}$, at the expense of computational time, it has been established that the influence of these smaller values on the contact pressure distribution is insignificant, so a predefined value of 10 µm is chosen in agreement with computational time. 

Although the developed contact mechanics model outputs the wear distribution and the other contact parameters uniformly in the face width direction, further development of the model is required [22,30]. Primarily, the changes in wear mechanism in the face width direction due to thermal effects need to be considered. Likewise, a detailed analysis of the surface roughness parameters, which can be implemented into the model, will most certainly lead to the non-uniform distribution of contact parameters. Additionally, the stiffness and thermal properties at the edge of the contact with corresponding alterations in wear distribution need to be investigated [13]. As the current input into the model is described with the theoretical involute profile, the model should also be adjusted to import geometric imperfections, manufacturing errors, and tooth modifications that have a great impact on the wear patterns and load distribution.

The case studies, presented for POM and PVDF gears with similar material and wear properties, reveal the behavior of the wear distribution and the corresponding changes in the contact parameters at a torque level of 5 Nm. In the initial phases of engagement, the maximum wear amounts are located at the start of the active profile, i.e., the tooth root zone, as shown in Figure 10a and Figure 15a, for POM and PVDF gears, respectively. The slight changes in the wear distribution are related to the established wear coefficients, presented in Table 4. At the same time, the maximum contact pressures are located at the same zones with the most intense wear mechanism, as shown in Figure 11a and Figure 16a, for POM and PVDF gears, respectively, with the differences in contact pressures that are related to material properties. Accordingly, the sliding conditions of POM and PVDF gears, presented in Figure 12a and Figure 17a, respectively, indicate the maximum values of sliding distances at the same zones. Even just by analyzing the initial phases of engagement, it is obvious that the active tooth profile, with the corresponding wear-induced changes in stiffness and normal load distribution, experiences the most severe contact pressures and sliding conditions in the areas with the most wear. As can be seen in the conducted case studies, the contact with the most intense wear mechanism, after the initial phases of engagement, gets shifted to the addendum area with a slight change in wear distribution at the pitch line zone, as presented in Figure 10b and Figure 15b, for POM and PVDF gears, respectively. Meanwhile, the previous areas with the most intense wear are reaching a wear-steady state, i.e., a limiting profile with approximately constant pressure distribution and sliding conditions, as shown in Figure 11b and Figure 12b for POM gears and Figure 16b and Figure 17b for PVDF gears. Similarly, in the late phases of engagement, presented in Figure 10c and Figure 15c, for POM and PVDF gears, respectively, the tooth tip areas are reaching a limiting profile with slow progressive wear at the pitch line zone, as notable in changes of contact pressure distribution and sliding conditions. These changes are presented in Figure 11c and Figure 12c for POM gears and Figure 16c and Figure 17c for PVDF gears. With the larger number of load cycles, the contact with the most intense wear is located around the pitch line, as shown in Figure 10d and Figure 15d, for POM and PVDF gears, respectively, with the limiting profile reached at the rest of the active profile. Accordingly, the maximum pressure distribution for POM and PVDF gears is located in the same area, as shown in Figure 11d and Figure 16d, respectively. The sliding distances with the maximum values correspond to the same area, as presented in Figure 12d and Figure 17d. Accordingly, these changes have a great influence on the degradation of meshing properties with the corresponding changes in the load-sharing factors, i.e., normal load distribution. 

When it comes to torque levels of 4 Nm and 6 Nm, with corresponding wear coefficients, a similar behavior in contact patterns can be expected, with the corresponding changes occurring at the different phases of engagement. A higher load level of 6 Nm will most certainly lead to a faster occurrence of the wear mechanism, i.e., tooth profile degradation and corresponding changes in contact parameters. Based on these data, it is possible to determine the functional lifespan of polymer gears regarding their wear characteristics, i.e., the maximum allowable wear depth, and to positively influence the design process or exploitation conditions so the polymer gears may be utilized most efficiently [31].

The developed model, as presented in this extensive study, results in a realistic wear prediction. Considering that the theory-based treatment of the sliding conditions at the pitch line zone, which results in zero sliding and zero wear due to pure rotation, differs from the real-life applications where polymer gears deflect as a result of lower and thermally dependent mechanical properties, the contact mechanics model considers this phenomenon by computing the sliding distances at the pitch line zone in consideration of the values of the sliding distances at the adjacent nodes. 

## 5. Conclusions

The main conclusions that can be drawn from the presented study are the following:

1. As the proposed contact mechanics model for wear prediction and experimentally obtained data are in good agreement, the presented model can be used for the wear prediction of parallel-axis polymer gears.
2. The model allows for the visualization and assessment of numerous contact parameters at different phases of engagement, i.e., number of load cycles under different load conditions, which can be very useful in the polymer gear design process and can reduce the need for time-consuming experimental tests.
3. The changes in contact parameters are altered with the changes in contact topography due to wear, with the maximum contact pressures and most severe sliding conditions at the most heavily worn areas of the tooth profile.

## Figures and Tables

**Table 1:** Basic geometric parameters of gears.

Parameter	Value
Module	3 mm
Number of teeth	17
Pressure angle	20°
Face width	20 mm

**Table 2:** POM material properties.

Parameter	Value	Test Method
Density	1.41 g/cm^3^	ISO 1183 [61]
Tensile modulus	2800 MPa	ISO 527-2 [62]
Tensile strength	67 MPa	ISO 527-2
Coefficient of linear expansion	1.4 × 10^−4^ K^−1^	ISO 11359 [63]
Thermal conductivity	0.39 W/(K⋅m)	ISO 22007-4 [64]
Melting temperature	166 °C	DIN 53765 [65]
Glass transition temperature	−85°	ISO 11375-1 [66]
Hardness (Brinell)	145 MPa	ISO 2039-1 [67]

**Table 3:** PVDF material properties.

Parameter	Value	Test Method
Density	1.78 g/cm^3^	ISO 1183
Tensile modulus	2000 MPa	ISO 527-2
Tensile strength	50 MPa	ISO 527-2
Coefficient of linear expansion	1.2 × 10^−4^ K^−1^	ISO 11359
Thermal conductivity	0.19 W/(K⋅m)	DIN 52612 [68]
Melting temperature	169 °C	ISO 3146 [69]
Glass transition temperature	−40°	DIN 53765
Hardness (Brinell)	90 MPa	ISO 2039-1

**Table 4:** Wear coefficients.

Engagement	Torque Level (Nm)		
	4	5	6
C45/POM	**Wear coefficient,** $k_{w}$ **(10^−6^ mm^3^/(Nm))**		
	5.58	9.77	12.96
C45/PVDF	**Wear coefficient,** $k_{w}$ **(10^−6^ mm^3^/(Nm))**		
	7.72	10.98	12.62

## Data Availability

The data presented in this study are available on request from the main author.
